# Tetra­kis(1-phenyl-1*H*-imidazole-κ*N* ^3^)bis­(thio­cyanato-κ*N*)nickel(II)

**DOI:** 10.1107/S1600536812015437

**Published:** 2012-04-18

**Authors:** Shao-Mei Zheng, Bao-Cheng Liu

**Affiliations:** a College of Mechanical Engineering, Qingdao Technological University, Qingdao 266033, People’s Republic of China; b Key Laboratory of Advanced Materials, Qingdao University of Science and Technology, Qingdao 266042, People’s Republic of China

## Abstract

The title compound, [Ni(NCS)_2_(C_9_H_8_N_2_)_4_], crystallizes with two independent half-mol­ecules in the asymmetric unit and the Ni^II^ ions situated on centres of symmetry. In both independent mol­ecules, the Ni^II^ ion displays a compressed octa­hedral environment formed by four N atoms from the 1-phenyl-1*H*-imidazole ligands, which define the equatorial plane, with a mean Ni—N distance of 2.119 (11) Å, and two axial N atoms from two NCS^−^ anions, with a mean Ni—N distance of 2.079 (7) Å. The crystal packing exhibits weak inter­molecular S⋯S contacts of 3.411 (2) Å.

## Related literature

For the crystal structures of related Ni complexes, see: Liu *et al.* (2005 ▶, 2006 ▶); Pang *et al.* (2007 ▶); Zheng & Jin (2012 ▶).
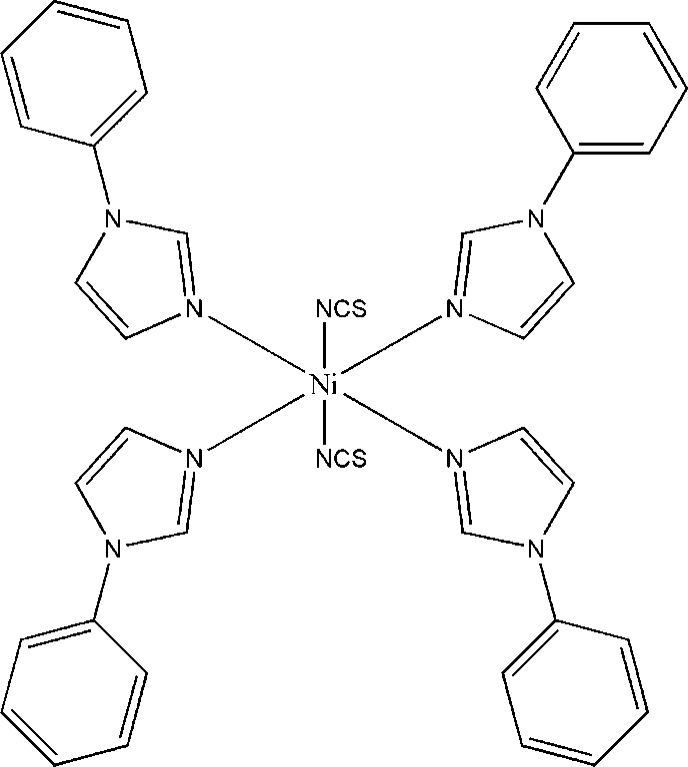


## Experimental

### 

#### Crystal data

[Ni(NCS)_2_(C_9_H_8_N_2_)_4_]
*M* *_r_* = 751.57
Triclinic, 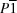

*a* = 9.9418 (5) Å
*b* = 12.8955 (6) Å
*c* = 16.7076 (8) Å
α = 68.239 (1)°
β = 77.563 (1)°
γ = 67.561 (1)°
*V* = 1831.91 (15) Å^3^
*Z* = 2
Mo *K*α radiation
μ = 0.69 mm^−1^
*T* = 293 K
0.32 × 0.31 × 0.19 mm

#### Data collection

Rigaku R-AXIS Spider diffractometer
Absorption correction: multi-scan (*ABSCOR*; Higashi 1995 ▶) *T*_min_ = 0.920, *T*_max_ = 0.936
15208 measured reflections
6791 independent reflections
4333 reflections with *I* > 2σ(*I*)
*R*_int_ = 0.040

#### Refinement

*R*[*F* ^2^ > 2σ(*F* ^2^)] = 0.062
*wR*(*F* ^2^) = 0.236
*S* = 1.12
6791 reflections
464 parameters
1 restraint
H-atom parameters constrained
Δρ_max_ = 0.76 e Å^−3^
Δρ_min_ = −1.30 e Å^−3^

### 

Data collection: *RAPID-AUTO* (Rigaku, 2004 ▶); cell refinement: *RAPID-AUTO*; data reduction: *RAPID-AUTO*; program(s) used to solve structure: *SHELXS97* (Sheldrick, 2008 ▶); program(s) used to refine structure: *SHELXL97* (Sheldrick, 2008 ▶); molecular graphics: *SHELXTL* (Sheldrick, 2008 ▶); software used to prepare material for publication: *SHELXTL*.

## Supplementary Material

Crystal structure: contains datablock(s) global, I. DOI: 10.1107/S1600536812015437/cv5267sup1.cif

Structure factors: contains datablock(s) I. DOI: 10.1107/S1600536812015437/cv5267Isup2.hkl

Supplementary material file. DOI: 10.1107/S1600536812015437/cv5267Isup3.cdx

Additional supplementary materials:  crystallographic information; 3D view; checkCIF report

## Figures and Tables

**Fig. 1.**
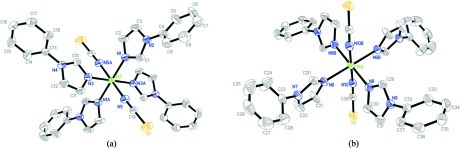
Two independent molecules of the title compound shown in (a) and (b), respectively, with 30% probability displacement ellipsoids and the atom-numbering scheme [symmetry codes: (A) -x+1, -y+2, -z; (B) -x+1, -y+1, -z+1]. H atoms were omitted for clarity.

## supplementary crystallographic information

### Comment

The title compound, (I), has been obtained in a study of the conditions of the formation of thiocyanate-containing complexes with imidazole derivatives, and to investigate the influence of steric properties on the stoichiometry as well as on the stoichiometry of the resulting species.

The asymmetric unit of (I) two independent half-molecules (Fig. 1). Each Ni atom displays an octahedral coordination geometry, with six N atoms from two thiocyanate anions and four 1-phenyl-1*H*-imidazole ligands. The equatorial plane of the complex is formed by four Ni—*N*(1-phenyl-1*H*-imadazole) bonds with lengths from 2.105 (4) to 2.127 (3) Å, and the axial positions are occupied by two N-bonded NCS groups [Ni—N(NCS) = 2.077 (4) or 2.083 (4) Å]. These values agree well with those observed in the related [Ni(NCS)_2_(1-methyl-1*H*-imidazole)_4_] (Liu *et al.*, 2005), [Ni(NCS)_2_(1-ethyl-1*H*-imidazole)_4_] (Liu *et al.*, 2006), [Ni(NCS)_2_(1-vinyl-1*H*-imidazole)_4_] (Pang *et al.*, 2007) and [Ni(NCS)_2_(1-allyl-1*H*-imidazole)_4_] (Zheng *et al.*, 2012). The values of the bond angles around nickel atoms are close to those expected for a regular octahedral geometry, the N—Ni—N angles range from 87.85 (13) to 92.15 (13) °, and the thiocyanate ligands are almost linear. Weak S···S intermolecular contacts of 3.411 (9) Å contribute to the crystal packing stability.

### Experimental

The title compound was prepared by the reaction of 1-phenyl-1*H*-imidazole (2.88 g, 20 mmol) with NiSO_4_.6H_2_O(1.31 g, 5 mmol) and potassium thiocyanate (0.98 g, 10 mmol) by means of hydrothermal synthesis in stainless-steel reactor with Teflon liner at 393 K for 24 h. Analysis, calculated for C_38_H_32_NiN_10_S_2_: C 60.73, H 4.29, N 18.64%; found: C 60.25, H 4.33, N 18.97%. Single crystals suitable for X-ray measurements were obtained by recrystallization from methanol at room temperature.

### Refinement

H atoms were positioned geometrically (C—H = 0.93–0.97 Å) and allowed to ride on their parent atoms with *U*_iso_(H) = 1.2 times *U*_eq_(C).

### Crystal data

**Table:**

[Ni(NCS)_2_(C_9_H_8_N_2_)_4_]	*Z* = 2
*M**_r_* = 751.57	*F*(000) = 780
Triclinic, *P*1	*D*_x_ = 1.362 Mg m^−^^3^
Hall symbol: -P 1	Mo *K*α radiation, λ = 0.71073 Å
*a* = 9.9418 (5) Å	Cell parameters from 9858 reflections
*b* = 12.8955 (6) Å	θ = 6.1–55.0°
*c* = 16.7076 (8) Å	µ = 0.69 mm^−^^1^
α = 68.239 (1)°	*T* = 293 K
β = 77.563 (1)°	Prism, blue
γ = 67.561 (1)°	0.32 × 0.31 × 0.19 mm
*V* = 1831.91 (15) Å^3^	

### Data collection

**Table:**

Rigaku R-AXIS Spider diffractometer	4333 reflections with *I* > 2σ(*I*)
Radiation source: fine-focus sealed tube	*R*_int_ = 0.040
Graphite monochromator	θ_max_ = 25.5°, θ_min_ = 3.1°
ω scans	*h* = −12→12
Absorption correction: multi-scan (*ABSCOR*; Higashi 1995)	*k* = −15→15
*T*_min_ = 0.920, *T*_max_ = 0.936	*l* = −18→20
15208 measured reflections	13 standard reflections every 0 reflections
6791 independent reflections	intensity decay: none

### Refinement

**Table:**

Refinement on *F*^2^	Secondary atom site location: difference Fourier map
Least-squares matrix: full	Hydrogen site location: inferred from neighbouring sites
*R*[*F*^2^ > 2σ(*F*^2^)] = 0.062	H-atom parameters constrained
*wR*(*F*^2^) = 0.236	*w* = 1/[σ^2^(*F*_o_^2^) + (0.1432*P*)^2^] where *P* = (*F*_o_^2^ + 2*F*_c_^2^)/3
*S* = 1.12	(Δ/σ)_max_ < 0.001
6791 reflections	Δρ_max_ = 0.76 e Å^−^^3^
464 parameters	Δρ_min_ = −1.30 e Å^−^^3^
1 restraint	Extinction correction: *SHELXL97* (Sheldrick, 2008), Fc^*^=kFc[1+0.001xFc^2^λ^3^/sin(2θ)]^-1/4^
Primary atom site location: structure-invariant direct methods	Extinction coefficient: 0.021 (3)

### Special details

**Table:**

Geometry. All e.s.d.'s (except the e.s.d. in the dihedral angle between two l.s. planes) are estimated using the full covariance matrix. The cell e.s.d.'s are taken into account individually in the estimation of e.s.d.'s in distances, angles and torsion angles; correlations between e.s.d.'s in cell parameters are only used when they are defined by crystal symmetry. An approximate (isotropic) treatment of cell e.s.d.'s is used for estimating e.s.d.'s involving l.s. planes.
Refinement. Refinement of *F*^2^ against ALL reflections. The weighted *R*-factor *wR* and goodness of fit *S* are based on *F*^2^, conventional *R*-factors *R* are based on *F*, with *F* set to zero for negative *F*^2^. The threshold expression of *F*^2^ > σ(*F*^2^) is used only for calculating *R*-factors(gt) *etc*. and is not relevant to the choice of reflections for refinement. *R*-factors based on *F*^2^ are statistically about twice as large as those based on *F*, and *R*- factors based on ALL data will be even larger.

### Fractional atomic coordinates and isotropic or equivalent isotropic displacement parameters (Å^2^)

**Table:**

	*x*	*y*	*z*	*U*_iso_*/*U*_eq_	
Ni1	0.5000	1.0000	0.0000	0.0460 (3)	
Ni2	0.5000	0.5000	0.5000	0.0442 (3)	
S1	0.8088 (2)	1.22650 (17)	−0.03145 (11)	0.0977 (6)	
S2	0.87351 (15)	0.13307 (12)	0.50231 (9)	0.0697 (4)	
N1	0.4373 (4)	0.9940 (3)	0.1310 (2)	0.0517 (9)	
N2	0.4097 (4)	1.0315 (3)	0.2539 (2)	0.0535 (9)	
N3	0.6640 (4)	0.8340 (3)	0.0386 (2)	0.0508 (9)	
N4	0.7766 (4)	0.6440 (3)	0.0980 (2)	0.0522 (9)	
N5	0.6440 (4)	1.0865 (3)	−0.0098 (2)	0.0570 (10)	
N6	0.5835 (4)	0.4575 (3)	0.6195 (2)	0.0492 (9)	
N7	0.6477 (4)	0.4845 (3)	0.7270 (2)	0.0540 (9)	
N8	0.6775 (4)	0.5613 (3)	0.4368 (2)	0.0507 (9)	
N9	0.8088 (4)	0.6767 (3)	0.3655 (2)	0.0495 (9)	
N10	0.6266 (5)	0.3303 (4)	0.4961 (2)	0.0553 (10)	
C1	0.4741 (5)	1.0472 (4)	0.1730 (3)	0.0550 (11)	
H1A	0.5374	1.0907	0.1491	0.066*	
C2	0.3436 (6)	0.9417 (5)	0.1898 (3)	0.0744 (16)	
H2A	0.2987	0.8971	0.1794	0.089*	
C3	0.3259 (6)	0.9637 (5)	0.2642 (3)	0.0745 (17)	
H3A	0.2676	0.9379	0.3136	0.089*	
C4	0.4229 (6)	1.0767 (5)	0.3165 (3)	0.0627 (13)	
C5	0.3001 (8)	1.1162 (6)	0.3697 (4)	0.095 (2)	
H5A	0.2099	1.1147	0.3640	0.114*	
C6	0.3158 (11)	1.1579 (8)	0.4311 (5)	0.126 (3)	
H6A	0.2351	1.1840	0.4676	0.151*	
C7	0.4482 (11)	1.1613 (7)	0.4391 (4)	0.118 (3)	
H7A	0.4570	1.1889	0.4812	0.141*	
C8	0.5664 (9)	1.1243 (7)	0.3858 (4)	0.103 (2)	
H8A	0.6558	1.1281	0.3904	0.124*	
C9	0.5537 (7)	1.0815 (5)	0.3253 (3)	0.0757 (16)	
H9A	0.6355	1.0552	0.2894	0.091*	
C10	0.6488 (5)	0.7313 (4)	0.0890 (3)	0.0524 (11)	
H10B	0.5598	0.7215	0.1148	0.063*	
C11	0.8119 (5)	0.8081 (4)	0.0162 (3)	0.0625 (13)	
H11A	0.8567	0.8632	−0.0186	0.075*	
C12	0.8822 (6)	0.6921 (5)	0.0516 (3)	0.0724 (15)	
H12A	0.9821	0.6525	0.0458	0.087*	
C13	0.8040 (5)	0.5196 (4)	0.1461 (3)	0.0528 (11)	
C14	0.8320 (7)	0.4408 (5)	0.1039 (3)	0.0716 (15)	
H14A	0.8309	0.4666	0.0441	0.086*	
C15	0.8622 (8)	0.3215 (5)	0.1504 (4)	0.0853 (18)	
H15A	0.8826	0.2668	0.1218	0.102*	
C16	0.8620 (7)	0.2846 (5)	0.2382 (4)	0.0805 (17)	
H16A	0.8806	0.2048	0.2697	0.097*	
C17	0.8346 (9)	0.3640 (6)	0.2797 (4)	0.099 (2)	
H17A	0.8369	0.3379	0.3395	0.118*	
C18	0.8030 (7)	0.4844 (5)	0.2339 (3)	0.0816 (17)	
H18A	0.7819	0.5392	0.2624	0.098*	
C19	0.7123 (5)	1.1451 (4)	−0.0195 (3)	0.0485 (10)	
C20	0.5666 (5)	0.5328 (4)	0.6586 (3)	0.0565 (11)	
H20A	0.5054	0.6112	0.6413	0.068*	
C21	0.6809 (7)	0.3545 (4)	0.6643 (3)	0.0770 (17)	
H21A	0.7148	0.2842	0.6512	0.092*	
C22	0.7211 (8)	0.3695 (5)	0.7305 (4)	0.0835 (18)	
H22A	0.7861	0.3126	0.7706	0.100*	
C23	0.6531 (5)	0.5427 (4)	0.7834 (3)	0.0541 (11)	
C24	0.5387 (8)	0.5762 (7)	0.8382 (5)	0.108 (3)	
H24A	0.4533	0.5614	0.8402	0.130*	
C25	0.5473 (11)	0.6321 (8)	0.8911 (6)	0.130 (3)	
H25A	0.4661	0.6567	0.9278	0.155*	
C26	0.6694 (10)	0.6526 (5)	0.8918 (4)	0.095 (2)	
H26A	0.6732	0.6894	0.9293	0.114*	
C27	0.7844 (9)	0.6199 (7)	0.8385 (6)	0.115 (3)	
H27A	0.8691	0.6343	0.8387	0.138*	
C28	0.7810 (6)	0.5645 (6)	0.7823 (5)	0.100 (2)	
H28A	0.8620	0.5424	0.7447	0.120*	
C29	0.6730 (5)	0.6682 (4)	0.3860 (3)	0.0504 (10)	
H29A	0.5880	0.7299	0.3667	0.061*	
C30	0.8223 (5)	0.4988 (4)	0.4487 (3)	0.0607 (12)	
H30A	0.8587	0.4195	0.4817	0.073*	
C31	0.9041 (5)	0.5680 (4)	0.4060 (3)	0.0622 (13)	
H31A	1.0049	0.5466	0.4042	0.075*	
C32	0.8492 (5)	0.7790 (4)	0.3110 (3)	0.0526 (11)	
C33	0.8203 (6)	0.8273 (5)	0.2259 (3)	0.0689 (14)	
H33A	0.7709	0.7971	0.2036	0.083*	
C34	0.8659 (7)	0.9215 (5)	0.1737 (3)	0.0780 (16)	
H34A	0.8490	0.9541	0.1155	0.094*	
C35	0.9360 (6)	0.9674 (5)	0.2076 (4)	0.0743 (15)	
H35A	0.9652	1.0317	0.1723	0.089*	
C36	0.9633 (6)	0.9190 (5)	0.2931 (4)	0.0701 (14)	
H36A	1.0109	0.9503	0.3157	0.084*	
C37	0.9201 (5)	0.8239 (4)	0.3453 (3)	0.0618 (13)	
H37A	0.9387	0.7904	0.4033	0.074*	
C38	0.7300 (5)	0.2473 (4)	0.4992 (3)	0.0470 (10)	

### Atomic displacement parameters (Å^2^)

**Table:**

	*U*^11^	*U*^22^	*U*^33^	*U*^12^	*U*^13^	*U*^23^
Ni1	0.0536 (5)	0.0521 (5)	0.0406 (4)	−0.0285 (4)	−0.0077 (3)	−0.0106 (3)
Ni2	0.0490 (5)	0.0441 (5)	0.0425 (4)	−0.0171 (4)	−0.0099 (3)	−0.0122 (3)
S1	0.1249 (14)	0.1269 (14)	0.0865 (11)	−0.0974 (13)	−0.0025 (9)	−0.0295 (10)
S2	0.0680 (9)	0.0588 (8)	0.0662 (8)	−0.0021 (7)	−0.0129 (6)	−0.0180 (6)
N1	0.060 (2)	0.061 (2)	0.0417 (19)	−0.031 (2)	−0.0054 (16)	−0.0134 (17)
N2	0.063 (2)	0.064 (2)	0.0433 (19)	−0.032 (2)	−0.0016 (16)	−0.0180 (17)
N3	0.060 (2)	0.061 (2)	0.0445 (19)	−0.031 (2)	−0.0075 (16)	−0.0173 (17)
N4	0.052 (2)	0.054 (2)	0.052 (2)	−0.023 (2)	−0.0026 (16)	−0.0127 (17)
N5	0.065 (2)	0.063 (2)	0.051 (2)	−0.032 (2)	−0.0123 (18)	−0.0110 (18)
N6	0.057 (2)	0.051 (2)	0.0442 (19)	−0.0210 (19)	−0.0116 (16)	−0.0129 (16)
N7	0.062 (2)	0.060 (2)	0.047 (2)	−0.021 (2)	−0.0144 (17)	−0.0183 (17)
N8	0.051 (2)	0.054 (2)	0.049 (2)	−0.0219 (19)	−0.0078 (16)	−0.0129 (17)
N9	0.053 (2)	0.053 (2)	0.0446 (19)	−0.0215 (19)	−0.0103 (16)	−0.0103 (16)
N10	0.064 (3)	0.058 (2)	0.048 (2)	−0.024 (2)	−0.0115 (18)	−0.0146 (18)
C1	0.062 (3)	0.064 (3)	0.045 (2)	−0.032 (3)	−0.005 (2)	−0.014 (2)
C2	0.095 (4)	0.102 (4)	0.051 (3)	−0.071 (4)	0.005 (3)	−0.018 (3)
C3	0.101 (4)	0.108 (4)	0.044 (3)	−0.073 (4)	0.010 (3)	−0.026 (3)
C4	0.083 (4)	0.071 (3)	0.044 (2)	−0.040 (3)	−0.002 (2)	−0.015 (2)
C5	0.105 (5)	0.129 (6)	0.080 (4)	−0.061 (5)	0.027 (4)	−0.063 (4)
C6	0.174 (8)	0.153 (7)	0.095 (5)	−0.084 (7)	0.052 (5)	−0.093 (5)
C7	0.206 (10)	0.133 (6)	0.068 (4)	−0.108 (7)	0.005 (5)	−0.046 (4)
C8	0.145 (7)	0.136 (6)	0.072 (4)	−0.090 (6)	−0.012 (4)	−0.035 (4)
C9	0.092 (4)	0.092 (4)	0.062 (3)	−0.044 (4)	−0.011 (3)	−0.028 (3)
C10	0.051 (3)	0.055 (3)	0.051 (2)	−0.021 (2)	−0.0046 (19)	−0.013 (2)
C11	0.061 (3)	0.062 (3)	0.065 (3)	−0.034 (3)	0.006 (2)	−0.013 (2)
C12	0.053 (3)	0.065 (3)	0.086 (4)	−0.026 (3)	0.006 (3)	−0.010 (3)
C13	0.058 (3)	0.052 (3)	0.049 (2)	−0.023 (2)	−0.001 (2)	−0.014 (2)
C14	0.103 (4)	0.062 (3)	0.056 (3)	−0.027 (3)	−0.020 (3)	−0.018 (2)
C15	0.127 (6)	0.062 (3)	0.082 (4)	−0.034 (4)	−0.018 (4)	−0.032 (3)
C16	0.102 (5)	0.055 (3)	0.075 (4)	−0.033 (3)	−0.002 (3)	−0.008 (3)
C17	0.150 (7)	0.081 (4)	0.049 (3)	−0.041 (4)	0.009 (3)	−0.009 (3)
C18	0.128 (5)	0.068 (3)	0.046 (3)	−0.038 (4)	0.002 (3)	−0.015 (2)
C19	0.055 (3)	0.060 (3)	0.041 (2)	−0.031 (2)	−0.0056 (18)	−0.0147 (19)
C20	0.062 (3)	0.058 (3)	0.053 (3)	−0.014 (2)	−0.019 (2)	−0.019 (2)
C21	0.121 (5)	0.045 (3)	0.070 (3)	−0.004 (3)	−0.062 (3)	−0.019 (2)
C22	0.125 (5)	0.052 (3)	0.074 (3)	−0.013 (3)	−0.053 (3)	−0.014 (3)
C23	0.067 (3)	0.057 (3)	0.047 (2)	−0.021 (2)	−0.016 (2)	−0.019 (2)
C24	0.107 (5)	0.155 (7)	0.117 (5)	−0.070 (5)	0.039 (4)	−0.103 (5)
C25	0.147 (8)	0.172 (9)	0.115 (6)	−0.071 (7)	0.032 (6)	−0.101 (6)
C26	0.143 (7)	0.074 (4)	0.078 (4)	−0.012 (4)	−0.048 (4)	−0.040 (3)
C27	0.094 (5)	0.142 (7)	0.159 (7)	−0.036 (5)	−0.030 (5)	−0.096 (6)
C28	0.078 (4)	0.145 (6)	0.126 (5)	−0.046 (4)	0.004 (4)	−0.093 (5)
C29	0.051 (3)	0.054 (3)	0.049 (2)	−0.021 (2)	−0.0088 (19)	−0.013 (2)
C30	0.057 (3)	0.058 (3)	0.063 (3)	−0.023 (3)	−0.015 (2)	−0.006 (2)
C31	0.050 (3)	0.064 (3)	0.068 (3)	−0.020 (3)	−0.014 (2)	−0.009 (2)
C32	0.052 (3)	0.058 (3)	0.049 (2)	−0.026 (2)	−0.0067 (19)	−0.010 (2)
C33	0.089 (4)	0.070 (3)	0.057 (3)	−0.038 (3)	−0.020 (3)	−0.012 (3)
C34	0.104 (4)	0.076 (4)	0.053 (3)	−0.041 (4)	−0.013 (3)	−0.003 (3)
C35	0.079 (4)	0.066 (3)	0.070 (3)	−0.035 (3)	−0.002 (3)	−0.005 (3)
C36	0.064 (3)	0.067 (3)	0.089 (4)	−0.035 (3)	−0.013 (3)	−0.019 (3)
C37	0.062 (3)	0.074 (3)	0.054 (3)	−0.032 (3)	−0.012 (2)	−0.013 (2)
C38	0.053 (3)	0.046 (2)	0.044 (2)	−0.018 (2)	−0.0114 (19)	−0.0108 (19)

### Geometric parameters (Å, º)

**Table:**

Ni1—N5^i^	2.074 (4)	C8—C9	1.366 (8)
Ni1—N5	2.074 (4)	C8—H8A	0.9300
Ni1—N3^i^	2.103 (4)	C9—H9A	0.9300
Ni1—N3	2.103 (4)	C10—H10B	0.9300
Ni1—N1	2.123 (3)	C11—C12	1.342 (7)
Ni1—N1^i^	2.123 (3)	C11—H11A	0.9300
Ni2—N10	2.084 (4)	C12—H12A	0.9300
Ni2—N10^ii^	2.084 (4)	C13—C14	1.356 (6)
Ni2—N8^ii^	2.121 (3)	C13—C18	1.364 (6)
Ni2—N8	2.121 (3)	C14—C15	1.386 (7)
Ni2—N6	2.129 (3)	C14—H14A	0.9300
Ni2—N6^ii^	2.129 (3)	C15—C16	1.364 (8)
S1—C19	1.611 (4)	C15—H15A	0.9300
S2—C38	1.605 (5)	C16—C17	1.355 (8)
N1—C1	1.326 (6)	C16—H16A	0.9300
N1—C2	1.372 (5)	C17—C18	1.394 (8)
N2—C1	1.343 (5)	C17—H17A	0.9300
N2—C3	1.368 (6)	C18—H18A	0.9300
N2—C4	1.421 (6)	C20—H20A	0.9300
N3—C10	1.326 (5)	C21—C22	1.354 (7)
N3—C11	1.376 (6)	C21—H21A	0.9300
N4—C10	1.330 (6)	C22—H22A	0.9300
N4—C12	1.371 (5)	C23—C24	1.337 (7)
N4—C13	1.448 (5)	C23—C28	1.3989 (10)
N5—C19	1.145 (5)	C24—C25	1.364 (9)
N6—C20	1.301 (6)	C24—H24A	0.9300
N6—C21	1.362 (6)	C25—C26	1.340 (11)
N7—C20	1.354 (6)	C25—H25A	0.9300
N7—C22	1.365 (7)	C26—C27	1.323 (10)
N7—C23	1.424 (5)	C26—H26A	0.9300
N8—C29	1.314 (5)	C27—C28	1.386 (9)
N8—C30	1.371 (6)	C27—H27A	0.9300
N9—C29	1.356 (5)	C28—H28A	0.9300
N9—C31	1.371 (6)	C29—H29A	0.9300
N9—C32	1.444 (5)	C30—C31	1.341 (6)
N10—C38	1.161 (6)	C30—H30A	0.9300
C1—H1A	0.9300	C31—H31A	0.9300
C2—C3	1.335 (7)	C32—C33	1.370 (6)
C2—H2A	0.9300	C32—C37	1.376 (6)
C3—H3A	0.9300	C33—C34	1.380 (7)
C4—C9	1.367 (8)	C33—H33A	0.9300
C4—C5	1.390 (8)	C34—C35	1.375 (8)
C5—C6	1.380 (9)	C34—H34A	0.9300
C5—H5A	0.9300	C35—C36	1.371 (7)
C6—C7	1.370 (11)	C35—H35A	0.9300
C6—H6A	0.9300	C36—C37	1.378 (6)
C7—C8	1.356 (11)	C36—H36A	0.9300
C7—H7A	0.9300	C37—H37A	0.9300
			
N5^i^—Ni1—N5	180.0 (2)	N3—C10—N4	111.6 (4)
N5^i^—Ni1—N3^i^	91.27 (14)	N3—C10—H10B	124.2
N5—Ni1—N3^i^	88.73 (14)	N4—C10—H10B	124.2
N5^i^—Ni1—N3	88.73 (14)	C12—C11—N3	110.4 (4)
N5—Ni1—N3	91.27 (14)	C12—C11—H11A	124.8
N3^i^—Ni1—N3	180.000 (1)	N3—C11—H11A	124.8
N5^i^—Ni1—N1	89.50 (13)	C11—C12—N4	105.9 (5)
N5—Ni1—N1	90.50 (13)	C11—C12—H12A	127.0
N3^i^—Ni1—N1	89.34 (14)	N4—C12—H12A	127.0
N3—Ni1—N1	90.66 (14)	C14—C13—C18	121.4 (5)
N5^i^—Ni1—N1^i^	90.50 (13)	C14—C13—N4	120.1 (4)
N5—Ni1—N1^i^	89.50 (14)	C18—C13—N4	118.4 (4)
N3^i^—Ni1—N1^i^	90.66 (14)	C13—C14—C15	119.6 (5)
N3—Ni1—N1^i^	89.34 (14)	C13—C14—H14A	120.2
N1—Ni1—N1^i^	180.0	C15—C14—H14A	120.2
N10—Ni2—N10^ii^	180.000 (1)	C16—C15—C14	119.8 (5)
N10—Ni2—N8^ii^	89.92 (14)	C16—C15—H15A	120.1
N10^ii^—Ni2—N8^ii^	90.08 (14)	C14—C15—H15A	120.1
N10—Ni2—N8	90.08 (14)	C17—C16—C15	120.1 (5)
N10^ii^—Ni2—N8	89.92 (14)	C17—C16—H16A	120.0
N8^ii^—Ni2—N8	180.0 (2)	C15—C16—H16A	120.0
N10—Ni2—N6	88.95 (14)	C16—C17—C18	120.8 (5)
N10^ii^—Ni2—N6	91.05 (14)	C16—C17—H17A	119.6
N8^ii^—Ni2—N6	92.21 (13)	C18—C17—H17A	119.6
N8—Ni2—N6	87.79 (13)	C13—C18—C17	118.3 (5)
N10—Ni2—N6^ii^	91.05 (14)	C13—C18—H18A	120.9
N10^ii^—Ni2—N6^ii^	88.95 (14)	C17—C18—H18A	120.9
N8^ii^—Ni2—N6^ii^	87.79 (13)	N5—C19—S1	179.0 (4)
N8—Ni2—N6^ii^	92.21 (13)	N6—C20—N7	112.7 (4)
N6—Ni2—N6^ii^	180.000 (1)	N6—C20—H20A	123.7
C1—N1—C2	104.0 (4)	N7—C20—H20A	123.7
C1—N1—Ni1	127.0 (3)	C22—C21—N6	110.0 (5)
C2—N1—Ni1	128.9 (3)	C22—C21—H21A	125.0
C1—N2—C3	105.9 (4)	N6—C21—H21A	125.0
C1—N2—C4	127.7 (4)	C21—C22—N7	106.6 (5)
C3—N2—C4	126.4 (4)	C21—C22—H22A	126.7
C10—N3—C11	104.6 (4)	N7—C22—H22A	126.7
C10—N3—Ni1	128.1 (3)	C24—C23—C28	118.9 (5)
C11—N3—Ni1	127.2 (3)	C24—C23—N7	121.5 (4)
C10—N4—C12	107.5 (4)	C28—C23—N7	119.6 (4)
C10—N4—C13	127.9 (3)	C23—C24—C25	120.0 (7)
C12—N4—C13	124.6 (4)	C23—C24—H24A	120.0
C19—N5—Ni1	172.7 (4)	C25—C24—H24A	120.0
C20—N6—C21	105.2 (4)	C26—C25—C24	122.0 (7)
C20—N6—Ni2	125.4 (3)	C26—C25—H25A	119.0
C21—N6—Ni2	128.8 (3)	C24—C25—H25A	119.0
C20—N7—C22	105.6 (4)	C27—C26—C25	119.3 (6)
C20—N7—C23	126.6 (4)	C27—C26—H26A	120.4
C22—N7—C23	127.8 (4)	C25—C26—H26A	120.4
C29—N8—C30	105.6 (3)	C26—C27—C28	121.2 (6)
C29—N8—Ni2	128.2 (3)	C26—C27—H27A	119.4
C30—N8—Ni2	126.0 (3)	C28—C27—H27A	119.4
C29—N9—C31	107.0 (3)	C27—C28—C23	118.6 (6)
C29—N9—C32	127.7 (4)	C27—C28—H28A	120.7
C31—N9—C32	125.4 (4)	C23—C28—H28A	120.7
C38—N10—Ni2	158.1 (3)	N8—C29—N9	111.0 (4)
N1—C1—N2	112.5 (4)	N8—C29—H29A	124.5
N1—C1—H1A	123.8	N9—C29—H29A	124.5
N2—C1—H1A	123.8	C31—C30—N8	110.4 (4)
C3—C2—N1	110.6 (4)	C31—C30—H30A	124.8
C3—C2—H2A	124.7	N8—C30—H30A	124.8
N1—C2—H2A	124.7	C30—C31—N9	106.1 (4)
C2—C3—N2	107.0 (4)	C30—C31—H31A	127.0
C2—C3—H3A	126.5	N9—C31—H31A	127.0
N2—C3—H3A	126.5	C33—C32—C37	121.2 (4)
C9—C4—C5	119.8 (5)	C33—C32—N9	119.7 (4)
C9—C4—N2	121.1 (5)	C37—C32—N9	119.1 (4)
C5—C4—N2	119.1 (5)	C32—C33—C34	118.9 (5)
C6—C5—C4	118.2 (7)	C32—C33—H33A	120.5
C6—C5—H5A	120.9	C34—C33—H33A	120.5
C4—C5—H5A	120.9	C35—C34—C33	120.2 (5)
C7—C6—C5	121.1 (7)	C35—C34—H34A	119.9
C7—C6—H6A	119.5	C33—C34—H34A	119.9
C5—C6—H6A	119.5	C36—C35—C34	120.4 (5)
C8—C7—C6	120.1 (6)	C36—C35—H35A	119.8
C8—C7—H7A	120.0	C34—C35—H35A	119.8
C6—C7—H7A	120.0	C35—C36—C37	119.8 (5)
C7—C8—C9	119.8 (7)	C35—C36—H36A	120.1
C7—C8—H8A	120.1	C37—C36—H36A	120.1
C9—C8—H8A	120.1	C32—C37—C36	119.4 (4)
C8—C9—C4	121.1 (6)	C32—C37—H37A	120.3
C8—C9—H9A	119.5	C36—C37—H37A	120.3
C4—C9—H9A	119.5	N10—C38—S2	179.3 (4)
			
N5^i^—Ni1—N1—C1	−171.8 (4)	C7—C8—C9—C4	1.0 (11)
N5—Ni1—N1—C1	8.2 (4)	C5—C4—C9—C8	0.2 (9)
N3^i^—Ni1—N1—C1	−80.5 (4)	N2—C4—C9—C8	−179.6 (5)
N3—Ni1—N1—C1	99.5 (4)	C11—N3—C10—N4	−0.9 (5)
N1^i^—Ni1—N1—C1	131 (100)	Ni1—N3—C10—N4	178.6 (3)
N5^i^—Ni1—N1—C2	3.7 (5)	C12—N4—C10—N3	0.6 (5)
N5—Ni1—N1—C2	−176.3 (5)	C13—N4—C10—N3	−178.5 (4)
N3^i^—Ni1—N1—C2	95.0 (4)	C10—N3—C11—C12	0.9 (6)
N3—Ni1—N1—C2	−85.0 (4)	Ni1—N3—C11—C12	−178.6 (4)
N1^i^—Ni1—N1—C2	−53 (100)	N3—C11—C12—N4	−0.6 (6)
N5^i^—Ni1—N3—C10	−29.2 (4)	C10—N4—C12—C11	0.0 (6)
N5—Ni1—N3—C10	150.8 (4)	C13—N4—C12—C11	179.1 (4)
N3^i^—Ni1—N3—C10	−123 (100)	C10—N4—C13—C14	103.0 (6)
N1—Ni1—N3—C10	60.3 (4)	C12—N4—C13—C14	−75.9 (6)
N1^i^—Ni1—N3—C10	−119.7 (4)	C10—N4—C13—C18	−78.0 (6)
N5^i^—Ni1—N3—C11	150.2 (4)	C12—N4—C13—C18	103.2 (6)
N5—Ni1—N3—C11	−29.8 (4)	C18—C13—C14—C15	−0.9 (9)
N3^i^—Ni1—N3—C11	56 (100)	N4—C13—C14—C15	178.1 (5)
N1—Ni1—N3—C11	−120.3 (4)	C13—C14—C15—C16	0.8 (10)
N1^i^—Ni1—N3—C11	59.7 (4)	C14—C15—C16—C17	−1.1 (10)
N5^i^—Ni1—N5—C19	62 (100)	C15—C16—C17—C18	1.5 (11)
N3^i^—Ni1—N5—C19	−11 (3)	C14—C13—C18—C17	1.3 (9)
N3—Ni1—N5—C19	169 (3)	N4—C13—C18—C17	−177.8 (6)
N1—Ni1—N5—C19	−101 (3)	C16—C17—C18—C13	−1.6 (11)
N1^i^—Ni1—N5—C19	79 (3)	Ni1—N5—C19—S1	128 (27)
N10—Ni2—N6—C20	169.4 (4)	C21—N6—C20—N7	−0.2 (6)
N10^ii^—Ni2—N6—C20	−10.6 (4)	Ni2—N6—C20—N7	−171.5 (3)
N8^ii^—Ni2—N6—C20	−100.7 (4)	C22—N7—C20—N6	0.0 (6)
N8—Ni2—N6—C20	79.3 (4)	C23—N7—C20—N6	−179.5 (4)
N6^ii^—Ni2—N6—C20	85 (100)	C20—N6—C21—C22	0.2 (7)
N10—Ni2—N6—C21	0.1 (4)	Ni2—N6—C21—C22	171.1 (4)
N10^ii^—Ni2—N6—C21	−179.9 (4)	N6—C21—C22—N7	−0.2 (7)
N8^ii^—Ni2—N6—C21	90.0 (4)	C20—N7—C22—C21	0.1 (6)
N8—Ni2—N6—C21	−90.0 (4)	C23—N7—C22—C21	179.7 (5)
N6^ii^—Ni2—N6—C21	−85 (100)	C20—N7—C23—C24	68.8 (7)
N10—Ni2—N8—C29	151.5 (4)	C22—N7—C23—C24	−110.7 (7)
N10^ii^—Ni2—N8—C29	−28.5 (4)	C20—N7—C23—C28	−111.8 (6)
N8^ii^—Ni2—N8—C29	−117 (100)	C22—N7—C23—C28	68.8 (7)
N6—Ni2—N8—C29	−119.6 (4)	C28—C23—C24—C25	0.8 (12)
N6^ii^—Ni2—N8—C29	60.4 (4)	N7—C23—C24—C25	−179.8 (7)
N10—Ni2—N8—C30	−36.2 (4)	C23—C24—C25—C26	−1.6 (15)
N10^ii^—Ni2—N8—C30	143.8 (4)	C24—C25—C26—C27	1.3 (15)
N8^ii^—Ni2—N8—C30	56 (100)	C25—C26—C27—C28	−0.2 (13)
N6—Ni2—N8—C30	52.8 (4)	C26—C27—C28—C23	−0.6 (13)
N6^ii^—Ni2—N8—C30	−127.2 (4)	C24—C23—C28—C27	0.3 (11)
N10^ii^—Ni2—N10—C38	19 (100)	N7—C23—C28—C27	−179.2 (6)
N8^ii^—Ni2—N10—C38	−142.1 (9)	C30—N8—C29—N9	−0.4 (5)
N8—Ni2—N10—C38	37.9 (9)	Ni2—N8—C29—N9	173.2 (3)
N6—Ni2—N10—C38	−49.9 (9)	C31—N9—C29—N8	0.1 (5)
N6^ii^—Ni2—N10—C38	130.1 (9)	C32—N9—C29—N8	179.6 (4)
C2—N1—C1—N2	0.0 (6)	C29—N8—C30—C31	0.6 (6)
Ni1—N1—C1—N2	176.4 (3)	Ni2—N8—C30—C31	−173.2 (3)
C3—N2—C1—N1	−0.1 (6)	N8—C30—C31—N9	−0.5 (6)
C4—N2—C1—N1	−179.2 (5)	C29—N9—C31—C30	0.3 (5)
C1—N1—C2—C3	0.1 (7)	C32—N9—C31—C30	−179.3 (4)
Ni1—N1—C2—C3	−176.2 (4)	C29—N9—C32—C33	−61.3 (7)
N1—C2—C3—N2	−0.1 (7)	C31—N9—C32—C33	118.1 (6)
C1—N2—C3—C2	0.1 (6)	C29—N9—C32—C37	120.6 (5)
C4—N2—C3—C2	179.3 (5)	C31—N9—C32—C37	−60.0 (7)
C1—N2—C4—C9	−39.1 (8)	C37—C32—C33—C34	1.1 (8)
C3—N2—C4—C9	142.0 (6)	N9—C32—C33—C34	−176.9 (5)
C1—N2—C4—C5	141.2 (6)	C32—C33—C34—C35	−1.5 (9)
C3—N2—C4—C5	−37.8 (8)	C33—C34—C35—C36	0.9 (10)
C9—C4—C5—C6	−0.9 (10)	C34—C35—C36—C37	0.0 (9)
N2—C4—C5—C6	178.8 (6)	C33—C32—C37—C36	−0.2 (8)
C4—C5—C6—C7	0.6 (12)	N9—C32—C37—C36	177.8 (5)
C5—C6—C7—C8	0.6 (14)	C35—C36—C37—C32	−0.3 (8)
C6—C7—C8—C9	−1.3 (13)	Ni2—N10—C38—S2	−87 (40)

Symmetry codes: (i) −*x*+1, −*y*+2, −*z*; (ii) −*x*+1, −*y*+1, −*z*+1.
